# Phase 1B/2 study of the HSP90 inhibitor AUY922 plus trastuzumab in metastatic HER2-positive breast cancer patients who have progressed on trastuzumab-based regimen

**DOI:** 10.18632/oncotarget.8974

**Published:** 2016-04-25

**Authors:** Anthony Kong, Daniel Rea, Samreen Ahmed, J. Thaddeus Beck, Rafael López López, Laura Biganzoli, Anne C. Armstrong, Massimo Aglietta, Emilio Alba, Mario Campone, Shu-Fang Hsu Schmitz, Caroline Lefebvre, Mikhail Akimov, Soo-Chin Lee

**Affiliations:** ^1^ Previous address: Churchill Hospital, Oxford University Hospitals NHS Trust and University of Oxford, Oxford, United Kingdom; ^2^ School of Cancer Sciences, University of Birmingham, Birmingham, United Kingdom; ^3^ Department of Oncology, University Hospitals of Leicester, Leicester Royal Infirmary, Leicester, United Kingdom; ^4^ Department of Oncology, Highlands Oncology Group, Fayetteville, Arkansas, USA; ^5^ Department of Oncology, Hospital Clinico Universitario, Santiago de Compostela, Spain; ^6^ Department of Medical Oncology, Nuovo Ospedale di Prato, Prato, Italy; ^7^ Department of Medical Oncology, The Christie NHS Foundation Trust, Manchester, United Kingdom; ^8^ Department of Medical Oncology, University of Torino, FPO-IRCCS, Candiolo, Italy; ^9^ Department of Medical Oncology, University Hospital, IBIMA, Malaga, Spain; ^10^ Department of Medical Oncology, Institut de Cancérologie de l'ouest René Gauducheau, Nantes, France; ^11^ Early Clinical Biostatistics, Oncology, Novartis Pharma AG, Basel, Switzerland; ^12^ Translational Clinical Oncology, Novartis Pharma AG, Basel, Switzerland; ^13^ Oncology Global Development, Novartis Pharma AG, Basel, Switzerland; ^14^ Department of Hematology-Oncology, National University Cancer Institute Singapore, Singapore

**Keywords:** breast cancer, HER2, HSP90 inhibitor, AUY922, trastuzumab

## Abstract

This open-label, multicenter, phase 1B/2 trial assessed AUY922 plus trastuzumab in patients with locally advanced or metastatic HER2-positive breast cancer previously treated with chemotherapy and anti-HER2 therapy. This study was composed of a dose-escalation part with AUY922 administered weekly at escalating doses with trastuzumab 2 mg/kg/week (phase 1B), followed by a phase 2 part using the same regimen at recommended phase 2 dose (RP2D). The primary objectives were to determine the maximum tolerated dose (MTD) and/or RP2D (phase 1B), and to evaluate preliminary antitumor activity (phase 2) of AUY922 plus trastuzumab at MTD/RP2D. Forty-five patients were treated with AUY922 plus trastuzumab (4 in phase 1B with AUY922 at 55 mg/m^2^ and 41 in phase 1B/2 with AUY922 at 70 mg/m^2^ [7 in phase 1B and 34 in phase 2]). One patient in phase 1B (70 mg/m^2^) experienced a dose-limiting toxicity (grade 3 diarrhea); the RP2D was weekly AUY922 70 mg/m^2^ plus trastuzumab. Of the 41 patients in the 70 mg/m^2^ cohort, the overall response rate (complete or partial responses) was 22.0% and 48.8% patients had stable disease. Study treatment-related adverse events occurred in 97.8% of patients; of these, 31.1% were grade 3 or 4. Forty-one patients (91.1%) reported ocular events (82.3% had grade 1 or 2 events). Two patients (4.4%) had ocular events leading to the permanent discontinuation of study treatment. AUY922 at 70 mg/m^2^ plus trastuzumab standard therapy is well tolerated and active in patients with HER2-positive metastatic breast cancer who progressed on trastuzumab-based therapy.

## INTRODUCTION

HER2 is overexpressed or amplified in approximately 15% to 20% of all cases of breast cancer [1]. Trastuzumab, a monoclonal anti-HER2 antibody, was the first biologically targeted therapy approved for the treatment of patients with advanced or metastatic HER2-positive breast cancer [2]. As a single agent, the overall response rates (ORR) of trastuzumab range between 12% and 26% [3–5]; ORR in patients with *HER2* overexpressed tumors based at the 3+ level by immunohistochemistry was 35% [5]. Trastuzumab monotherapy improved disease-free survival in patients with HER2-positive breast cancer in the adjuvant setting (eg, HERceptin Adjuvant [HERA] trial) [6].

Trastuzumab has been used as the first-line treatment in combination with chemotherapy such as docetaxel or paclitaxel in locally advanced or metastatic HER2-positive breast cancer, with response rates ranging between 50% and 80% [2, 7] and as the second-line or later treatment in combination with different cytotoxic agents [ORR, 20%-68%] [8–16]. The majority of patients with metastatic breast cancer who initially responded to trastuzumab developed resistance within 1 year of treatment initiation [17].

Lapatinib (a dual inhibitor of epidermal growth factor receptor [EGFR] tyrosine kinase 1 and 2 [HER2]) in combination with capecitabine has been shown to prolong progression-free survival (PFS), and this combination is indicated for the treatment of patients with advanced or metastatic HER2-positive breast cancer who have received prior therapy including an anthracycline, a taxane, and trastuzumab [18].

Pertuzumab (a HER2-targeting antibody) when combined with trastuzumab and docetaxel as a first-line treatment prolonged overall survival (OS) in patients with HER2-positive metastatic breast cancer [19]. Trastuzumab–maytansine conjugate (TDM-1, a HER2 antibody-drug conjugate) significantly improves PFS in previously treated patients with metastatic HER2-positive breast cancer [20, 21]. Pertuzumab and TDM-1 have been approved as first-line and second-line treatments for use in metastatic breast cancer on June 08, 2012 and February 22, 2013, respectively.

HSP, a family of the molecular chaperones, are a group of proteins that play essential role in the folding of a large number of cellular proteins [22, 23]. HSP90 interacts with a variety of proteins that play important roles in breast cancer including receptor tyrosine kinases such as EGFR and HER2 as well as RAF and AKT [24].

The first-generation compounds of HSP90 inhibitors were geldanamycin derivatives, which have a few limitations including difficult formulation and hepatotoxicity [25–27]. The new, second-generation, synthetic HSP90 inhibitors such as AUY922 have greater potency and reduced hepatotoxicity. AUY922 is an isoxazole-based, nongeldanamycin compound, which competitively inhibits the ATPase activity of HSP90, resulting in degradation of client proteins, including HER2 [28]. AUY922 has shown activity in preclinical breast cancer model, and significant synergy was observed when it was combined with trastuzumab in the HER-2 positive BT-474 breast cancer xenograft model [28]. A first-in-human phase 1 study of AUY922 in advanced solid tumors showed that patients tolerated weekly infusions of AUY922, and dose-limiting toxicities (DLTs) occurred in 8 patients (7.9%) (22-70 mg/m^2^), which included diarrhea, asthenia/fatigue, anorexia, atrial flutter, and visual symptoms. The recommended phase 2 dose (RP2D) of AUY922 was declared as 70 mg/m^2^ once weekly [29].

Clinical data of the HSP90 inhibitor tanespimycin in combination with trastuzumab have shown promising activity in patients with HER2-positive locally advanced or metastatic breast cancer progressing on trastuzumab [30]. Ganetespib, another second-generation HSP90 inhibitor has shown promising clinical activity (clinical benefit rate of 60%) in combination with paclitaxel and trastuzumab (clinical benefit rate was 60%) in heavily pretreated patients with HER2-positive metastatic BC [31]. This open-label, multicenter, phase 1B/2 trial evaluated the efficacy, safety, tolerability, biologic activity, and pharmacokinetic profile of AUY922 in combination with trastuzumab in patients with locally advanced or metastatic HER2-positive breast cancer previously treated with chemotherapy and anti-HER2 therapy.

## RESULTS

### Baseline characteristics and patient disposition

A total of 45 patients with HER2-positive advanced or metastatic breast cancer (median age, 51.0 years [range, 29.0-71.0]) were treated with AUY922 plus trastuzumab. Of these 45 patients, 88.9% of patients had invasive ductal carcinoma, 4.4% of patients had lobular carcinoma, and 6.7% of patients had carcinoma of other histology; 55.6% of patients were estrogen receptor (ER)-positive and 42.2% of patients were ER-negative; 40.0% of patients were progesterone receptor (PR)-positive, and 57.8% of patients were PR-negative; 1 patient (2.2%) had missing data for both ER and PR status; 11.1%, 26.7%, and 62.2% of patients had received 1, 2, and ≥ 3 regimens at last treatment, respectively (Table 1). The trial recruited the first patient in September 2010, and the last patient's recruitment was completed on May 2012. During this time, pertuzumab and TDM-1 were not approved as anti-HER2 treatments (see Introduction for the dates of approval). However, patients who received either of these agents were eligible for the trial. In total, 3 (6.7%) and 8 patients (17.8%) received prior TDM-1 and pertuzumab, respectively. A total of 12 patients (26.7%) had received prior lapatinib treatment.

**Table 1:** Patient demographics and disease characteristics

Characteristic, n (%)	55 mg/m^2^ n = 4	70 mg/m^2^ n = 41	All patients N = 45
Median age (range), years	53.5 (43.0, 64.0)	51.0 (29.0, 71.0)	51.0 (29.0, 71.0)
Age category, years			
< 65	4 (100)	36 (87.8)	40 (88.9)
≥ 65	0	5 (12.2)	5 (11.1)
Race			
Caucasian	2 (50.0)	34 (82.9)	36 (80.0)
Black	0	1 (2.4)	1 (2.2)
Asian	2 (50.0)	6 (14.6)	8 (17.8)
Histology			
Invasive ductal carcinoma	3 (75.0)	37 (90.2)	40 (88.9)
Invasive lobular carcinoma	0	2 (4.9)	2 (4.4)
Other	1 (25.0)	2 (4.9)	3 (6.7)
Estrogen receptor-negative	4 (100)	15 (36.6)	19 (42.2)
Estrogen receptor-positive	0	25 (61.0)	25 (55.6)
Missing	0	1 (2.4)	1 (2.2)
Progesterone receptor-negative	4 (100)	22 (53.7)	26 (57.8)
Progesterone receptor-positive	0	18 (43.9)	18 (40.0)
Missing	0	1 (2.4)	1 (2.2)
Stage at diagnosis			
I to IIb	4 (100)	20 (48.8)	24 (53.3)
III to IIIb	0	14 (34.1)	14 (31.1)
IV	0	5 (12.2)	5 (11.1)
Missing	0	2 (4.9)	2 (4.4)
Metastatic sites			
Brain	0	1 (2.4)	1 (2.2)
Pleura	0	2 (4.9)	2 (4.4)
Lung	2 (50.0)	22 (53.7)	24 (53.3)
Liver	0	23 (56.1)	23 (51.1)
Pancreas	0	1 (2.4)	1 (2.2)
Peritoneum	0	1 (2.4)	1 (2.2)
Breast	1 (25.0)	4 (9.8)	5 (11.1)
Uterus	0	1 (2.4)	1 (2.2)
Kidney	0	2 (4.9)	2 (4.4)
Skin	1 (25.0)	2 (4.9)	3 (6.7)
Bone	0	25 (61.0)	25 (55.6)
Lymph nodes	2 (50.0)	11 (26.8)	13 (28.9)
Other	0	4 (9.8)	4 (8.9)
Number of regimens at last treatment			
1	0	5 (12.2)	5 (11.1)
2	2 (50.0)	10 (24.4)	12 (26.7)
≥ 3	2 (50.0)	26 (63.4)	28 (62.2)
Therapy type at last medication			
Chemotherapy	2 (50.0)	24 (58.5)	26 (57.8)
Hormonal therapy	0	7 (17.1)	7 (15.6)
Targeted therapy	4 (100)	29 (70.7)	33 (73.3)
Prior antineoplastic therapy – monoclonal antibodies			
Trastuzumab	4 (100)	41 (100)	45 (100)
Trastuzumab emtansine (TDM-1)	1 (25.0)	2 (4.9)	3 (6.7)
Bevacizumab	0	2 (4.9)	2 (4.4)
Pertuzumab	2 (50.0)	6 (14.6)	8 (17.8)
Lapatinib	1 (25.0)	11 (26.8)	12 (26.7)

Four patients were treated in phase 1B with AUY922 at 55 mg/m^2^ plus weekly trastuzumab and 41 patients were treated in phase 1B/2 with AUY922 at 70 mg/m^2^ plus trastuzumab (7 patients in phase 1B and 34 patients in phase 2). As of January 29, 2013, forty-three patients discontinued the treatment; the majority of patients (68.9%) discontinued the treatment due to disease progression (Table 2). Follow-up was completed in 42 out of 43 patients who had discontinued treatment.

**Table 2:** Patient disposition

Patient disposition	55 mg/m^2^ n = 4	70 mg/m^2^ n = 41	All patients N = 45
Patients enrolled, n (%)			
Treated	4 (100)	41 (100)	45 (100)
Treatment discontinued	4 (100)	39 (95.1)	43 (95.6)
Treatment ongoing	0	2 (4.9)	2 (4.4)
Primary reason for end of treatment, n (%)			
Adverse event(s)	1 (25.0)	7 (17.1)	8 (17.8)
Subject withdrew consent	0	4 (9.8)	4 (8.9)
Disease progression	3 (75.0)	28 (68.3)	31 (68.9)

### Determination of maximum tolerated dose (MTD)/RP2D

In the first-in-human AUY922 monotherapy study, the RP2D was declared as 70 mg/m^2^ once weekly [29]. In this study, only 1 patient in the phase1B (70 mg/m^2^ cohort) experienced a DLT (grade 3 diarrhea) with trastuzumab. The RP2D was thus weekly AUY922 70 mg/m^2^ in combination with trastuzumab.

### Efficacy

**Figure 1:**
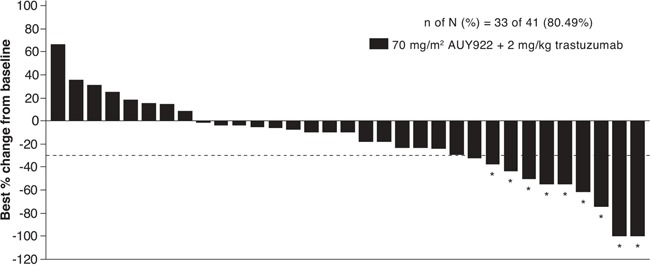
Best tumor shrinkage (computed tomography response) in target lesions among evaluable patients in the 70 mg/m^2^ cohort *Confirmed Response Evaluation Criteria in Solid Tumors response. ‘n’ represents number of patients with a baseline and at least 1 post-baseline tumor assessment in target lesions by investigator and ‘N’ represents total number of patients treated with AUY922 70 mg/m^2^ plus trastuzumab.

**Figure 2:**
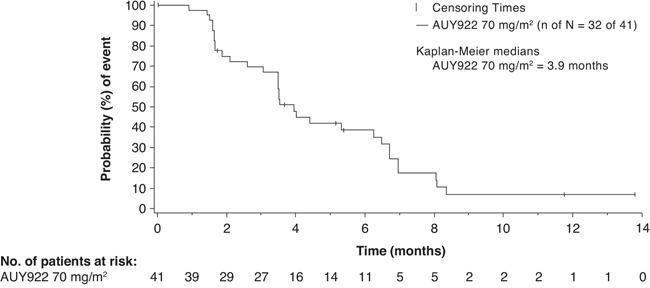
Kaplan–Meier plot of progression-free survival as per investigator in the 70 mg/m^2^ cohort ‘n’ represents number of patients with progressive disease or death whereas ‘N’ represents total number of patients treated with AUY922 70 mg/m^2^ plus trastuzumab. Patients who have not had events are censored, but are still included in the figure.

**Figure 3:**
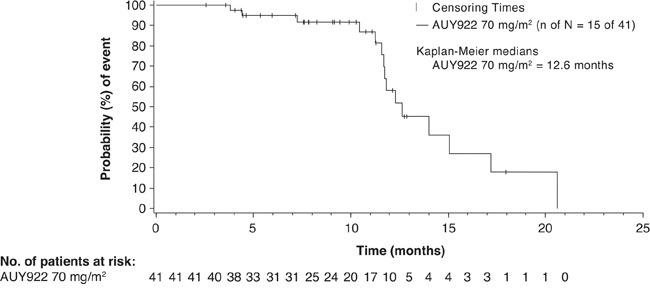
Kaplan–Meier plot of overall survival as per investigator in the 70 mg/m^2^ cohort ‘n’ represents number of deaths whereas ‘N’ represents total number of patients treated with AUY922 70 mg/m2 plus trastuzumab. Patients who have not died are censored, but are still included in the figure.

Among the 41 patients in the 70 mg/m^2^ cohort, the ORR (complete or partial responses) by investigator review was 22.0% (Table 3). From the Bayesian posterior distribution, the mean ORR was 22.0% [95% credible interval: 11.1%, 35.5%]. The best tumor shrinkage (computed tomography [CT] response) in target lesions in evaluable patients is shown in Figure 1. Twenty patients (48.8%) had stable disease. The median PFS was 3.94 months (95% confidence interval [CI]: 3.48, 6.47) (Figure 2). The Kaplan-Meier estimates of PFS rate at 4 and 6 months were 47.9% (95% CI: 31.4, 62.7) and 38.7% (95% CI: 23.1, 54.1), respectively. The median OS was 12.65 months (95% CI: 11.70, 17.22) (Figure 3). The Kaplan-Meier estimate of OS rate at 8 months was 91.6% (95% CI: 76.0, 97.2).

**Table 3:** Best overall response by investigator review

Best overall response, n (%)	70 mg/m^2^ n = 41
Complete response (CR)	1 (2.4)
Partial response (PR)	8 (19.5)
Stable disease	20 (48.8)
Progressive disease	11 (26.8)
Unknown	1 (2.4)
Objective response (CR or PR), n (%) [95% credible interval]	9 (22.0) (11.1, 35.5)

### Pharmacokinetics

**Figure 4:**
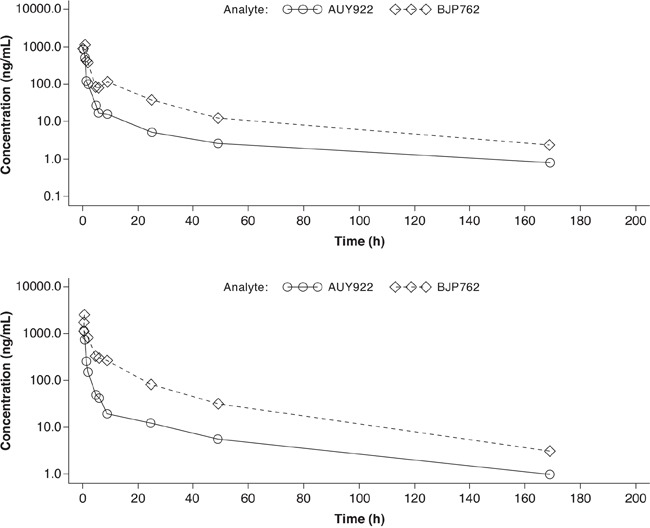
Semi-logarithmic arithmetic mean concentration-time profiles for plasma AUY922 and BJP762 in combination with trastuzumab **A.** AUY922 55 mg/m^2^ plus trastuzumab. **B.** AUY922 70 mg/m^2^ plus trastuzumab.

Mean plasma concentration-time profiles for AUY922 and its metabolite BJP762 in combination with trastuzumab on cycle 1 day 1 were both biphasic at doses of 55 mg/m^2^ and 70 mg/m^2^ (Figure 4A and Figure 4B, respectively).

The exposures to AUY922 and BJP762 in plasma for the 70 mg/m^2^ dose cohort were generally higher than those in the 55 mg/m^2^ cohort, indicating dose-dependent increase in exposure. AUY922 had a mean clearance (CL) value of 60 L/h (61.50 L/h for 55 mg/m^2^ cohort and 59.27 L/h for 70 mg/m^2^ cohort) and a mean volume of distribution (Vz) value of 3850 L (4541.60 L for 55 mg/m^2^ cohort and 3160.83 L for 70 mg/m^2^ cohort) in plasma.

### Safety

The median duration of treatment (AUY922 plus trastuzumab) exposure was 16.0 weeks (range, 1-72) in all patients; the median duration of treatment in the 55 mg/m^2^ and 70 mg/m^2^ cohorts was 10.5 weeks (range, 7-24) and 16.0 weeks (range, 1-72), respectively.

Of the overall 45 patients treated with AUY922 plus trastuzumab, 37.8% patients (17 of 45) had ≥ 1 dose reduction (50.0% [2 of 4] in the 55 mg/m^2^ cohort and 36.6% [15 of 41] in the 70 mg/m^2^ cohort). All of the dose reductions were due to adverse events (AEs), except for 2 patients in the 70 mg/m^2^ cohort (1 due to dosing error and 1 due to a laboratory test abnormality). Twenty-six patients had ≥ 1 dose delay (2 in the 55 mg/m^2^ cohort and 24 in the 70 mg/m^2^ cohort). Overall, 46.7% patients (21 of 45) had dose delays due to AEs (50.0% in the 55 mg/m^2^ cohort [2 of 4] and 46.3% in the 70 mg/m^2^ cohort [19 of 41]).

All patients reported ≥ 1 AE, regardless of study treatment relationship; the most common AEs were diarrhea (88.9%), fatigue (37.8%), nausea (37.8%), headache (31.1%), night blindness (31.1%), and visual impairment (31.1%). Overall, 21 patients (46.7%) experienced a grade 3 or 4 AE regardless of study treatment relationship; the most common grade 3 AEs were dyspnea, (3 patients, 6.7%) and accommodation disorder, anemia, arthralgia, diarrhea, decreased ejection fraction, fatigue, and nausea (in 2 patients each, 4.4%). Six patients in the 70 mg/m^2^ cohort experienced grade 4 AEs regardless of causality (anemia, hyperkalemia, jaundice, optic nerve disorder, pleural effusion, and breast cancer [2 events in 1 patient]).

Study treatment-related AEs occurred in 97.8% patients; the most frequent AEs were diarrhea, fatigue, nausea, and visual impairment (Table 4). Study treatment-related grade 3 or 4 AEs occurred in 14 patients (31.1%). The most common study-treatment related grade 3 or 4 AEs were diarrhea, fatigue, accommodation disorder, and decreased ejection fraction (each occurring in 2 patients, 4.4%).

**Table 4:** Study treatment-related adverse events (all grades ≥ 10% and grades 3 or 4)

Preferred term, n (%)	55 mg/m^2^ n = 4		70 mg/m^2^ n = 41		All patients N = 45	
	All grades	Grade 3 or 4	All grades	Grade 3 or 4	All grades	Grade 3 or 4
Diarrhea	2 (50.0)	0	38 (92.7)	2 (4.9)	40 (88.9)	2 (4.4)
Fatigue	2 (50.0)	0	13 (31.7)	2 (4.9)	15 (33.3)	2 (4.4)
Nausea	1 (25.0)	0	14 (34.1)	1 (2.4)	15 (33.3)	1 (2.2)
Visual impairment	2 (50.0)	1 (25.0)	12 (29.3)	0	14 (31.1)	1 (2.2)
Night blindness	1 (25.0)	0	12 (29.3)	0	13 (28.9)	0
Photopsia	3 (75.0)	0	10 (24.4)	1 (2.4)	13 (28.9)	1 (2.2)
Vision blurred	2 (50.0)	0	11 (26.8)	0	13 (28.9)	0
Vitreous floaters	1 (25.0)	0	10 (24.4)	0	11 (24.4)	0
Accommodation disorder	1 (25.0)	0	9 (22.0)	2 (4.9)	10 (22.2)	2 (4.4)
Asthenia	0	0	8 (19.5)	1 (2.4)	8 (17.8)	1 (2.2)
Decreased appetite	0	0	7 (17.1)	0	7 (15.6)	0
Headache	1 (25.0)	0	6 (14.6)	0	7 (15.6)	0
Vomiting	1 (25.0)	0	6 (14.6)	1 (2.4)	7 (15.6)	1 (2.2)
Abdominal pain	0	0	6 (14.6)	0	6 (13.3)	0
Muscle spasms	0	0	6 (14.6)	0	6 (13.3)	0
Pruritus	1 (25.0)	0	5 (12.2)	0	6 (13.3)	0
Anemia	0	0	5 (12.2)	0	5 (11.1)	0
Hypokalemia	0	0	5 (12.2)	0	5 (11.1)	0
Photophobia	2 (50.0)	0	3 (7.3)	0	5 (11.1)	0
Visual acuity reduced	0	0	5 (12.2)	0	5 (11.1)	0

Overall, 11 patients (24.4%) (all in the 70 mg/m^2^ cohort) experienced serious AEs (SAEs), regardless of causality; none of these resulted in discontinuation of study treatment or death. Three patients (7.3%) in the 70 mg/m^2^ cohort experienced study treatment-related SAEs; none of which resulted in permanent discontinuation.

Eight patients (1 patient in the 55 mg/m^2^ cohort and 7 patients in the 70 mg/m^2^ cohort) (17.8%) discontinued the treatment due to AEs, regardless of study treatment relationship. Study treatment-related discontinuation events occurred in 7 patients, which included elevated alanine aminotransferase, hyperkalemia, optic nerve disorder, decreased appetite, accommodation disorder (each in 1 patient); and decreased ejection fraction (2 patients).

A total of 18 deaths occurred > 28 days after the last dose and during the survival follow-up period (data cutoff, January 29, 2013). The cause of death was reported as disease progression in 16 patients; 1 patient died during brain surgery, and 1 due to acute respiratory insufficiency.

Overall, 41 patients (91.1%) reported ocular events regardless of study treatment relationship (all 4 patients in the 55 mg/m^2^ cohort and 37 patients in the 70 mg/m^2^ cohort); the most frequent ocular events were night blindness (31.1%), visual impairment (31.1%), photopsia (28.9%), blurred vision (28.9%), and photophobia (11.1%). Most ocular events (82.3%) were grade 1 or 2. Four patients (8.9%) (1 in the 55 mg/m^2^ cohort and 3 in the 70 mg/m^2^ cohort) experienced grade 3 or 4 ocular events; all were study treatment related. Two patients (4.4%) had ocular events (grade 4 optic nerve disorder and grade 3 accommodation disorder) leading to permanent discontinuation of study treatment, both considered to be study treatment related.

One patient in the 70 mg/m^2^ cohort (2.4% of this cohort) experienced 2 cardiac events of interest (left bundle branch block and cardiac failure). Both were SAEs of grade 3 severity and were suspected to be related to study drug; neither led to permanent discontinuation of study treatment.

## DISCUSSION

This phase 1B/2 study evaluated the efficacy and safety of AUY922 plus trastuzumab in patients with advanced or metastatic HER-2 positive breast cancer progressing on trastuzumab. The rationale for this study was based on synergistic activity of AUY922 plus trastuzumab in the HER-2 positive BT-474 breast cancer xenograft model [28] and clinical experience of other HSP90 inhibitors in combination with trastuzumab in patients with advanced HER2-positive metastatic breast cancer progressing on trastuzumab [30].

In phase 1B, only 1 patient (70 mg/m^2^ cohort) experienced a DLT (grade 3 diarrhea) in combination with trastuzumab. According to the Bayesian logistic regression model, at a 70 mg/m^2^ dose of AUY922, the posterior risk of DLT rate being in the excessive toxicity category was much lower than 25%; hence, the dose of AUY922 could have been further escalated. However, at that time, the AUY922 first-in-human monotherapy study [29] had already declared 70 mg/m^2^ as the RP2D; thus, further escalation was prohibited. The dose of 70 mg/m^2^ weekly was then taken as the RP2D in combination with weekly trastuzumab.

In this study, 22.0% of patients treated at 70 mg/m^2^ achieved ORR by investigator review. Furthermore, stable disease was reported in 48.8% of patients treated at 70 mg/m^2^. Thus, our results confirm that HSP90 inhibition in combination with trastuzumab is a promising strategy in advanced or metastatic HER2-positive breast cancer patients progressing on trastuzumab. The results are comparable to that seen with tanespimycin plus trastuzumab combination, where ORR was 22% and disease stabilization rate was 37% [30]. In the first-in-human monotherapy study of AUY922, which included metastatic breast cancer [29], none of the patients had objective responses; this may be due to the following 2 reasons: the patients in the first-in-human study were not molecularly prioritized before study entry; and the responses seen in this study may be due to the combination of trastuzumab with AUY922. Despite the promising ORR and disease stabilization rate, the median PFS and OS were only 3.94 months and 12.65 months, respectively.

The trough trastuzumab concentrations following administration of trastuzumab were higher than 30 μg/mL, which is consistent with the observations reported in the literature [32]. The pharmacokinetic profile of AUY922 in combination with trastuzumab seen in this study is consistent with that observed in the AUY922 first-in-human monotherapy study [29].

The safety profile of AUY922 plus trastuzumab was similar to the safety profile of AUY922 monotherapy in the first-in-human study with the most common AEs being gastrointestinal and ocular [29]. Of note, a majority of patients (82.3%) had grade 1 or 2 ocular AEs; grade 3 and grade 4 ocular AEs were reported in 3 (6.7%) and 1 patients (2.2%), respectively. Overall, only 2 patients discontinued the treatment due to ocular events; other ocular events (mostly ≥ grade 2) were managed by study treatment dosage adjustment/temporary interruption and/or use of concomitant medication. However, ocular events seen in this study were not unexpected, as it has been reported in the first-in-human AUY922 monotherapy study where ocular events related to AUY922 occurred in 43% of patients (grade 1 or 2 in 41 of 101 patients [41%] and grade 3 in 2 of 101 patients [2%]). Of note, ocular events were not reported in HER2-positive breast cancer patients treated with tanespimycin [30]. The difference in ocular toxicity in AUY922 and tanespimycin may be due to high retina/plasma concentration ratio and slow elimination profile of AUY922 [33].

The anti-HER2 paradigms for metastatic breast cancer have changed since the initiation of this study; pertuzumab is approved in combination with trastuzumab and docetaxel as first-line treatment for patients with HER2-positive metastatic breast cancer who have not received prior anti-HER2 therapy or chemotherapy for metastatic disease, whereas TDM-1 is approved for patients with HER2-positive metastatic breast cancer previously treated with trastuzumab.

In conclusion, the combination of AUY922 at 70 mg/m^2^ and trastuzumab standard therapy is well tolerated and active in patients with HER2-positive metastatic breast cancer who progressed on trastuzumab-based therapy; however, this needs to be explored further in a larger population. Although there are no clinical studies ongoing with AUY922 in metastatic breast cancer, this study has provided further rationale for combining HSP90 inhibitor with trastuzumab in pretreated HER2-positive breast cancer patients.

## PATIENTS AND METHODS

### Patients

Female patients, who were aged ≥ 18 years with confirmed HER2-positive, nonoperable, locally advanced or metastatic breast cancer who progressed on prior anti–HER2-based regimens including at least 1 regimen containing trastuzumab, were eligible. HER2 overexpression was based on either immunohistochemistry at the 3+ level or immunohistochemistry 2+ level confirmed by fluorescence *in situ* hybridization.

Patients who developed metastases while receiving adjuvant trastuzumab were eligible, and their adjuvant therapy was considered as 1 prior regimen.

Patients who received lapatinib as a last line of treatment were eligible. Other key inclusion criteria were ≥ 1 measurable lesion as defined by Response Evaluation Criteria In Solid Tumors version 1.0 (RECIST), documented progressive disease following the last line of therapy, and Eastern Cooperative Oncology Group performance status of ≤ 1.

Patients were excluded if they had the following: known symptomatic central nervous system metastases requiring treatment (for symptom control and/or growing); impaired cardiac function; received prior treatment with any HSP90 or histone deacetylase inhibitor; systemic anticancer treatment prior to AUY922 dosing (radiotherapy, chemotherapy, hormonotherapy, investigational drugs, and monoclonal antibodies other than trastuzumab within 4 weeks; palliative radiotherapy within 2 weeks; and nitrosoureas and mitomycin within 6 weeks); unresolved diarrhea > Common Toxicity Criteria for Adverse Events version 4.0 (CTCAE) grade 1; not recovered from the reversible side effects of previous systemic anticancer therapy (except for alopecia) < CTCAE grade 2 prior to the first dose; therapeutic doses of sodium warfarin; acute or chronic liver or renal disease; other concurrent severe and/or uncontrolled medical conditions that could cause unacceptable safety risks; known hypersensitivity to any study medication; and history of another primary malignancy that was currently clinically significant or currently required active intervention.

The study protocol and all amendments were reviewed by the Independent Ethics Committee or Institutional Review Board for each center. The study was conducted according to Good Clinical Practice guidelines and ethical principles of the Declaration of Helsinki. All patients provided written informed consent.

### Study design and treatment

This open-label, nonrandomized, multicenter study (NCT01271920) was composed of a dose-escalation part with AUY922 administered at escalating doses in combination with trastuzumab at the standard dose (phase 1B), followed by a phase 2 part using the same regimen with AUY922 and trastuzumab at RP2D. AUY922 was administered as once weekly 1-hour intravenous infusion with trastuzumab at the licensed weekly dose of 2 mg/kg over 30 minutes (or 4 mg/kg over 90 minutes if a loading dose was necessary at cycle 1 day 1) on days 1, 8, 15, and 22 of each 28-day treatment cycle. Patients received treatment until disease progression, unacceptable toxicity, or investigator's/patient's decision to discontinue the treatment.

A 2-parameter Bayesian logistic regression model employing the escalation with overdose control (EWOC) principle [34, 35] was used during the dose-escalation phase to guide dose-level selection and determination of the MTD/RP2D. The starting dose of AUY922 was 55 mg/m^2^ in phase 1B and was determined based on EWOC criteria and data from first-in-human AUY922 monotherapy study [29]. The primary objective for phase 1B was to determine the MTD and/or RP2D of AUY922 plus trastuzumab, whereas the primary objective of the phase 2 component was to evaluate preliminary antitumor activity (ORR) of AUY922 plus trastuzumab at the MTD/RP2D. Secondary objectives include evaluation of pharmacokinetics, safety, tolerability, and preliminary efficacy (PFS, OS) for AUY922 in combination with trastuzumab standard therapy.

### Patient assessments

Safety assessments consisted of monitoring by investigators and recording all AEs and SAEs (up to 28 days following the last dose of AUY922), and the regular monitoring of laboratory evaluations, physical examination, vital signs, weight, performance status evaluation, electrocardiograms and repeat cardiac assessments, cardiac enzymes, echocardiogram and or multigated acquisition scan (if clinically indicated). Ophthalmologic examinations were also to be performed at the end of cycle 2 and end of treatment or more often if any visual symptom was reported. Toxicity was assessed using the National Cancer Institute CTCAE, version 4.0.

All patients were to be followed for AEs and SAEs for 28 days following the last dose of AUY922. Pharmacokinetic parameters were determined by noncompartmental method(s) using WinNonlin^®^ Pro (version 5.2). Tumor response was assessed by the investigators according to RECIST version 1.0 [36]. Tumor assessments using CT or magnetic resonance imaging were to be performed every 8 weeks for first 24 weeks and every 12 weeks thereafter until progression or until a new anticancer therapy was initiated. The best overall response of SD was defined as having at least one SD assessment (or better) > 6 weeks after start of treatment (and not qualifying for CR or PR).

### Statistical methods

The EWOC principle [34, 35] mandated that any dose of AUY922 in combination with the standard trastuzumab therapy that had more than a 25% chance of being in the excessive toxicity category (true DLT rate in the interval [33%, 100%]) was not considered for the next dose cohort. Prior distributions for the Bayesian logistic regression model parameters were based on data from the AUY922 first-in-human study [29].

For phase 1B, 3 to 6 patients evaluable for the Bayesian logistic regression model were to be treated per cohort, until the MTD/RP2D was reached, and ≥ 6 patients evaluable for the dose-determining set were to be treated at the MTD/RP2D. With the use of historical data from the AUY922 first-in-human study [29] in an informative prior distribution, it was estimated that approximately 12 patients would need to be enrolled on study for the model to have reasonable operating characteristics.

The phase 2 part used a Bayesian design to estimate the ORR observed with this regimen with AUY922 at the identified MTD and/or RP2D. For phase 2 part, given an observed ORR equal to 30%, approximately 40 patients (including those already treated at the RP2D during the phase 1B part) were planned for the model to have less than 10% posterior risk of the true response rate being less than 20%.

PFS and OS were estimated by Kaplan-Meier method.
